# Isolation and Characterization of a Lytic Vibriophage OY1 and Its Biocontrol Effects Against *Vibrio* spp.

**DOI:** 10.3389/fmicb.2022.830692

**Published:** 2022-04-07

**Authors:** Lu Gao, Min Ouyang, Yi Li, Hui Zhang, Xiang-Feng Zheng, Hua-Xiang Li, Sheng-Qi Rao, Zhen-Quan Yang, Song Gao

**Affiliations:** ^1^College of Food Science and Engineering, Yangzhou University, Yangzhou, China; ^2^Key Laboratory of Prevention and Control of Biological Hazard Factors (Animal Origin) for Agri-food Safety and Quality, Ministry of Agriculture, Yangzhou, China; ^3^Jiangsu Key Laboratory for Food Quality and Safety-State Key Laboratory Cultivation Base, Ministry of Science and Technology, Nanjing, China; ^4^College of Veterinary Medicine, Yangzhou University, Yangzhou, China; ^5^Jiangsu Co-innovation Center for Prevention and Control of Important Animal Infectious Diseases and Zoonoses, Yangzhou University, Yangzhou, China

**Keywords:** vibriophage, lytic activity, biocontrol, *Vibrio* species, biofilm

## Abstract

*Vibrio* species are important pathogens of marine animals and aquaculture populations and some of them can cause serious infections in humans through consumption of contaminated seafood and aquaculture products. Lytic bacteriophages can potentially alleviate *Vibrio* contamination in the aquaculture organisms and in the processing of aquatic products and have gained significant scientific attention in recent years. In the present study, bacteriophages were isolated from sewage of local aquatic products markets and grown using *Vibrio mimicus* CICC 21613 as host cells. The lytic vibriophage OY1 belonging to the newly proposed family *Autographiviridae* and the genus *Maculvirus* was identified by observation under electron microscope and comparative genomic analysis. The phage OY1 showed lytic activity against 24 among 32 tested strains belonging to eight *Vibrio* species. The complete phage OY1 genome consists of a single circular double-stranded DNA of 43,479 bp with a total GC content of 49.27% and was predicted to encode 40 open reading frames (ORFs). To evaluate its potential against vibrios, the one-step growth curve, thermal and pH stability, host range, and lytic activity of the OY1 phage against *Vibrio* species were evaluated. The results showed that phage OY1 had a range of thermal and pH tolerance, and exhibited a significant inhibitory effect on the growth of tested *Vibrio* species. Bacterial growth in the fish muscle extract juice (FMEJ) inoculated with *Vibrio mimicus* CICC 21613, *Vibrio parahaemolyticus* CICC 21617, *Vibrio alginolyticus* VJ14, and the mixed bacterial culture was reduced by 2.65 log CFU/ml, 2.42 log CFU/ml, 1.93 log CFU/ml, and 2.01 log CFU/ml, respectively, by incubation with phage OY1 at 25°C for 36 h. Phage OY1 also showed a strong ability to prevent biofilm formation and destroy formed *Vibrio* species biofilms. These results indicate that phage OY1 is a potential biocontrol agent against *Vibrio* species in the aquaculture industry and in food safety control.

## Introduction

*Vibrio* species are a group of genetically and metabolically diverse Gram-negative motile bacteria distributed in various habitats, especially in marine, brackish, and freshwater ecosystems (Kalatzis et al., 2018). The high abundance and versatile features allow *Vibrio* species to play important roles in biogeochemical cycles of aquatic ecosystems (Thompson et al., 2004). Pathogenic *Vibrio* species are responsible for vibriosis in aquaculture populations and marine animals (Wong et al., 1995; Lee et al., 1996; Anguiano-Beltrán et al., 1998; Austin and Zhang, 2006; Wan et al., 2011; Hong et al., 2016; Dabu et al., 2017). Some *Vibrio* species, e.g., *Vibrio alginolyticus*, *V. cholerae*, *V. cincinnatiensis*, *V. damsela*, *V. fluvialis*, *V. furnissii*, *V. harveyi*, *V. metschnikovii*, *V. mimicus*, *V. parahaemolyticus*, and *V. vulnificus* are known human pathogens (Davis et al., 1981; Brenner et al., 1983; Wachsmuth et al., 1994; Finkelstein et al., 2002; Jones and Oliver, 2009). Due to their high abundance in marine and aquaculture populations, various types of seafood, reared fish, and shellfish may be contaminated by *Vibrio* species (Roxana et al., 2010; Charles and Anthony, 2017), and consumption of these aquatic products can cause gastroenteritis and lead to disease outbreaks (Chitov et al., 2009; Geng et al., 2014; Osunla and Okoh, 2017).

Controlling approaches against *Vibrio* infections in humans and in the aquaculture industry include vaccination, administration of antibiotics, and application of lytic vibriophages (Nicolás et al., 2018). Vaccination has been quite successful in controlling *Vibrio* infections in fish (Buchmann et al., 2001; Fouz et al., 2001), but unfortunately, no commercial vaccines are available against most of the pathogenic *Vibrio* species other than *V. anguillarum* (Kalatzis et al., 2018). Administration of antibiotics is currently the most commonly used strategy to control vibriosis. However, the use and abuse of antibiotics not only disturbs the natural microbiota of various ecosystems (Li et al., 1999), but also leads to spread of antibiotic-resistant bacteria in the environment, causing significant public health problems (Chowdhury et al., 1986).

The ever-increasing side effects of use of vaccines and antibiotics to control pathogenic *Vibrio* species has led to exploration of new alternatives. Utilizing lytic bacteriophages as biocontrol agents was thus proposed and considered as one of the most promising options (Clark, 2015), not only for treatment, but also for prevention of *Vibrio* infections. Bacteriophages are usually specific to particular host bacterial strains or certain species; they will not remain in the environment in the absence of the host, without disturbing other microorganisms. The use of lytic bacteriophages against pathogenic *Vibrio* strains has increased the survival rates of several cultured animals in experimental aquaculture setups (reviewed in Kalatzis et al., 2018), providing better protection in some cases compared to antibiotics (Karunasagar et al., 2007; Zhang et al., 2015). Better performances were achieved in response to a phage cocktail treatment rather than using individual phages (Mateus et al., 2014; Li et al., 2016; Doss et al., 2017). Besides the aquaculture industry, bacteriophages have also been used as biocontrol agents in the food industry (Endersen et al., 2014). For instance, phage preparation ListShield™ (LMP-102™), developed by Intralytix Inc., United States, and Listex P100 developed by EBI Food Safety, the Netherlands, were approved by the US Food and Drug Administration (FDA) for use as a processing aid in foods susceptible to contamination by *Listeria monocytogenes* (Linda, 2007; Hagens and Offerhaus, 2008). The use of anti-*E. coli* and anti-*Salmonella* phage-based preparations were also approved to reduce contamination during meat processing (Sillankorva et al., 2004; Goodridge and Bisha, 2011). Various studies have shown that lytic bacteriophages can be excellent alternatives to control pathogenic *Vibrio* species in aquaculture and marine organisms (Lomelí-Ortega and Martínez-Díaz, 2014; Letchumanan et al., 2016; Nicolás et al., 2018). However, this approach has not been widely used and is still being explored (Plaza et al., 2018). No phage-based products are commercialized at present. Several constraints of phage therapy, such as efficacy, delivery method, persistence of phages under field conditions, phage resistance, and unwanted phage-encoded products (e.g., toxins), should be addressed before a phage therapy application can be considered successful (Kalatzis et al., 2018).

The present study reports the isolation of a podovirus bacteriophage OY1 which showed lytic activity against certain strains from six *Vibrio* species. The phage OY1 was identified to belong to the newly proposed family *Autographiviridae* and the genus *Maculvirus* by a combination of morphological and comparative genomic characterization. The thermal and pH stability, host range, and lytic activity of the phage in liquid culture and in fish muscle extract juice (FMEJ) were determined. The phage’s ability to prevent biofilm formation and to destroy biofilms was explored to evaluate its potential as a biocontrol agent against *Vibrio* species using both singly and the mixed cultures of three isolates, i.e., *V. mimicus* CICC 21613, *V. parahaemolyticus* CICC 21617, and *V. alginolyticus* VJ14. In addition, the annotated genome of the phage was compared with the three closely related *Maculvirus* phage species.

## Materials and Methods

### Bacterial Strains and Culture Conditions

A total of 32 bacterial strains from 11 *Vibrio* species were used in this study (Table 1). Six of these strains were obtained from the China Center of Industrial Culture Collection (CICC), two from the China General Microbiological Culture Collection Center (CGMCC), and the other strains were isolated from either aquatic products or clinical isolates from human fecal samples. Strains isolated in this study were identified by 16S rRNA gene sequencing. Stock strains were maintained at −80°C. All the bacteria were grown at 37°C on lysogeny broth (LB; Sigma Aldrich, United Kingdom) supplemented with 3% (w/v) NaCl (3% LB).

**Table 1:** *Vibrio* spp. strains used in this study and the host range test of phage OY1.

Species	Strains	Origin	EOP
*V. aestuarianus*	VI13	Fish/Jiangsu, China	5.7 × 10^−3^ ± 0.3 × 10^−3^
*V. alginolyticus*	CICC 10484	Food/Japan	0
	VF221	Shrimp/Jiangsu, China	2.3 × 10^−1^ ± 0.8 × 10^−2^
	VE13	Shellfish/Jiangsu, China	4.9 × 10^−5^ ± 0.3 × 10^−5^
	VJ14	Crab/Jiangsu, China	8.7 × 10^−1^ ± 0.7 × 10^−2^
	VH23	Fish/Jiangsu, China	0
	VG23	Fish/Jiangsu, China	7.2 × 10^−2^ ± 0.4 × 10^−3^
	VD31	Shrimp/Jiangsu, China	3.6 × 10^−4^ ± 0.7 × 10^−4^
*V. azureus*	VJ31	Fish/Jiangsu, China	1.7 × 10^−2^ ± 0.1 × 10^−2^
	VC32	Shrimp/Jiangsu, China	0
	VT44	Shrimp/Jiangsu, China	1.8 × 10^−2^ ± 0.9 × 10^−3^
	VK34	Fish/Jiangsu, China	5.7 × 10^−3^ ± 0.3 × 10^−3^
*V. cholerae*	CICC 23794	China	0
*V. fluvialis*	CGMCC 1.1609	Unknown	0
	CICC 21612	Faeces/Bangladesh	3.2 × 10^−7^ ± 0.8 × 10^−7^
*V. harveyi*	CGMCC 1.1609	Seawater/California, United States	0
*V. metschnikovii*	VN42	Fish/Jiangsu, China	4.3 × 10^−1^ ± 0.5 × 10^−2^
	VM44	Crab/Jiangsu, China	0
*V. mimicus*	CICC 21613	Human/Clinical isolate/United States	1
	CICC 10474	China	2.6 × 10^−3^ ± 0.3 × 10^−3^
*V. natriegens*	VL22	Fish/Jiangsu, China	4.7 × 10^−6^ ± 0.8 × 10^−6^
*V. owensii*	VH51	Crab/Jiangsu, China	0
*V. parahaemolyticus*	CICC 21617	Food/Japan	9.1 × 10^−1^ ± 0.4 × 10^−2^
	Vp 68	Human/Clinical isolate/China	7.7 × 10^−1^ ± 0.6 × 10^−2^
	Vp 76	Human/Clinical isolate/China	1.3 × 10^−1^ ± 0.7 × 10^−2^
	Vp 92	Human/Clinical isolate/China	3.8 × 10^−2^ ± 0.1 × 10^−2^
	Vp 101	Human/Clinical isolate/China	8.5 × 10^−3^ ± 0.4 × 10^−3^
	Vp 110	Human/Clinical isolate/China	2.7 × 10^−2^ ± 0.1 × 10^−2^
	VJ34	Fish/Jiangsu, China	8.3 × 10^−1^ ± 0.2 × 10^−2^
	VF13	Crab/Jiangsu, China	5.1 × 10^−3^ ± 0.1 × 10^−3^
	VI52	Shellfish/Jiangsu, China	6.4 × 10^−2^ ± 0.3 × 10^−2^
	VD23	Shrimp/Jiangsu, China	4.3 × 10^−3^ ± 0.8 × 10^−4^

*EOP, efficiency of plaquing was calculated (average PFU on target bacteria/average PFU on *V. mimicus* CICC 21613) along with the SD for the three measurements. “0,” indicate bacterial strain was not susceptible to phage OY1*.

### Phage Isolation, Purification, and Propagation

Six sewage samples (100 ml each) were collected from local markets trading in aquatic products in Yangzhou, Jiangsu, China. Thirty milliliters of each sample was centrifuged at 8,000 *g* (Eppendorf 5810R, Hamburg, Germany) for 10 min to remove the solid impurities and was filtered using 0.22 μm Millex® filters (Merck Millipore Ltd. of Tullagreen, Carrigtwohill, Co., Cork, Munster, Ireland). Each filtrate (5 ml) was added to an equal volume of 2% × 3% LB, inoculated with 0.1 ml of a mid-exponential phase culture of host bacteria *V. mimicus* CICC 21613, and incubated at 37°C with overnight shaking at 150 rpm. For each sample, the bacteria were removed by centrifugation (8,000 *g* for 10 min at 4°C) and the supernatant was passed through a 0.22 μm pore size filter. The filtrate was collected for each sample and the presence of phages was confirmed using the spot test as described by Yang et al. (2019).

### Morphological Observation

Each phage stock sample with a titer of ~10^9^ PFU/ml was purified by density gradient centrifugation using CsCl gradients (Sambrook et al., 1989). Twenty microliters of purified phage (~10^11^ PFU/ml) was deposited onto carbon-coated cuprum grids and allowed to adsorb for 10 min. The phage particles were then negatively stained with 2% (w/v) potassium phosphotungstate (pH 7.2), and examined under a Tecnai 12 transmission electron microscope (Philips Electron Optics, Eindhoven, The Netherlands).

### Host Range Test

The host range of phage OY1 was tested following the method described in Yang et al. (2019). Thirty-two *Vibrio* strains belong to 11 species were used (Table 1), and three independent experiments were performed.

### One-Step Growth Curve and Lytic Activity Against *Vibrio mimicus* in Liquid Culture

To determine the one-step growth curve of the phage, 100 μl of the phage suspension with a titer of ~10^9^ PFU/ml was mixed with an equal volume of the exponential phase culture of *V. mimicus* CICC 21613 (~10^8^ CFU/ml) in 5 ml of 3% LB pre-warmed at 37°C and allowed to adsorb for 10 min. The mixture was centrifuged at 8,000 × *g* for 5 min. The cell pellets were washed three times with 3% LB and re-suspended in 5 ml of 3% LB that been pre-warmed to 37°C. This suspension was incubated at the same temperature with shaking at 150 rpm. Samples (100 μl) were taken every 10 up to 120 min, and phage titers were determined by the double-layered agar plate method. One-step growth curves were plotted and the latent period, rise period, and phage burst size of OY1 were calculated as previously described (Pujato et al., 2015).

Lytic activity of phage OY1 in liquid culture against *V. mimicus* CICC 21613 was tested in 3% LB. One milliliter of phage OY1 suspension with a titer of ~10^9^ PFU/ml was mixed with 1 ml exponential phase host bacterial culture (~10^6^ CFU/ml) and 8 ml 3% LB, equivalent to an approximate multiplicity of infection (MOI) of 1,000, and incubated at 37°C with shaking. In negative control, an equal volume of 3% LB was added instead of the phages. The liquid cultures were sampled and the absorbance was measured at 600 nm every 2 h.

### Thermal and pH Stability

For thermal stability, 0.5 ml of phage suspension was incubated at 50°C, 60°C, 70°C, and 80°C, and aliquots were taken every 20 min to determine the phage titers by the double-layer agar method using *V. mimicus* CICC 21613. For pH stability, 100 μl of the phage suspension (~10^9^ PFU/ml) was added into 5 ml of 3% LB at different pH values (pH 2.0–12.0, adjusted using NaOH or HCl), incubated at 37°C for 2 h, and then titers were calculated immediately by the double-layer agar method using *V. mimicus* CICC 21613.

### DNA Extraction, Genome Sequencing, and Annotation

The purified phage suspension used for genomic DNA extraction was prepared following protocols as described by Yang et al. (2019). The incubation temperature of host bacteria was modified to 37°C (optimal temperature of *V. mimicus* CICC 21613). Phage DNA extraction and purification were performed using the ABigen Lambda Phage DNA Purification kit (AB1141, ABigen Corporation, Beijing, China). Total DNA was visualized by 1% agarose gel electrophoresis and quantified with a QuBit DNA quantification system (Invitrogen). A 500 bp DNA library was constructed and 150 bp paired end (PE) sequencing was carried out on an Illumina HiSeq system at Total Genomics Solution (TGS), Shenzhen, China. Data trimming, genome assembly, and annotation were performed by TGS. A total of 1,263 Mb of clean data was trimmed out from 1,481 Mb of the raw sequencing output. Repetitive sequences were identified using RepeatMasker ver. 3.3.0^1^ (Saha et al., 2008) and Tandem Repeats Finder (TRF) ver. 4.04^2^ (Benson, 1999). Open reading frame (ORFs) encoding hypothetical proteins were predicted using Prodigal software version 2.6.3 (Hyatt et al., 2010). Translated ORF sequences were compared with known proteins using standard protein–protein BLASTP^3^ (Altschul and Lipman, 1990) against five databases including the GenBank Non-Redundant Protein Sequence Database (NRDB), Kyoto Encyclopedia of Genes and Genomes (KEGG), Clusters of Orthologous Groups of proteins (COG), Gene Ontology (GO) resource and the SWISS-PROT. An online standard nucleotide BLAST against NCBI (National Center for Biotechnology Information) database was performed using DNA sequences of predicted open reading frames (ORFs) as queries, and predictions at *E*-value <10^−5^ were adopted. The genetic map was generated with Circos software (Krzywinski et al., 2009) and modified by Adobe Illustrator® CS5 (Version 15.0.0, Adobe®, San Jose, CA, United States). The annotated genome was deposited in GenBank under the accession number OM799543.

### Lytic Activity of Phage OY1 in FMEJ

FMEJ was used as a model to determine the lytic activity of phage OY1 for its potential as a biocontrol agent for *Vibrio* species. FMEJ was prepared using large yellow croaker species as described by Yang et al. (2019). Three bacterial strains, i.e., *V. mimicus* CICC 21613, *V. parahaemolyticus* CICC 21617, and *V. alginolyticus* VJ14, which showed the highest EOP value for each species during the host range test, finally were used. Five hundred microliters of exponential phase growth bacterial cultures (~10^7^ CFU/ml) of each strain was mixed with FMEJ (3% NaCl), inoculated with 500 μl of phage suspensions (~10^9^ PFU/ml), and then incubated at 25°C. FMEJ cultures were sampled at 2, 4, 6, 8, 12, and 16 h post-inoculation. The log values of colony-forming units (CFU) of the culture samples were determined, and the same three bacterial strains in FMEJ culture without phage inoculation were used as control. The lytic activity of phage OY1 against a mixed bacterial culture comprising 167 μl (~10^7^ CFU/ml) of each strain was also tested.

### Ability of Phage OY1 to Prevent Biofilm Formation and to Destroy Biofilms

Static microtiter plate assay was used to evaluate the ability of phage OY1 to prevent biofilm formation and to destroy biofilms. *V. mimicus* CICC 21613, *V. parahaemolyticus* CICC 21617, *V. alginolyticus* VJ14, and the mixed bacterial culture (containing an equal volume of each bacterium) were used. Overnight bacterial cultures were suspended in 3% LB to get ~10^7^ CFU/ml cultures. For each strain, an equal volume of the bacterial cultures (100 μl) and the phage suspension (100 μl containing ~10^9^ PFU/ml) was added into wells of sterile 96-well plates and incubated at 37°C for 24 h. After the incubation, the liquid culture was discarded and the wells were washed three times with 200 μl of sterilized phosphate-buffered saline (PBS) buffer (150 mM NaCl, 10 mM Na_2_HPO_4_/NaH_2_PO_4_, pH 7.4) to remove the planktonic cells. The plates were dried at room temperature (25°C) and then stained with 200 μl of 0.2% crystal violet solution for 30 min. The wells were washed three times with PBS to remove the excess dye and dried at room temperature. The biofilms formed were dissolved with 33% glacial acetic acid. The ability of the phage OY1 to prevent biofilm formation was assessed by measuring the OD_600 nm_ of the dissolved solution. For testing the biofilm destruction, the phage suspension was added after the incubation of bacteria at 37°C for 24 h and treated for 4 h under the same temperature; the blank control used 3% LB. Bacterial inoculation without phage suspension was also used as a positive control.

### Statistical Analysis

Statistical analyses were performed with the SPSS statistical software package version 16.0 (SPSS Inc., Chicago, Illinois, United States). The differences between treatment and control groups were determined by Student’s *t*-test. Results with *p* < 0.01 (the confidence level is 99%) were considered statistically significant.

## Results

### Isolation and Morphological Characterization of Phage OY1

**Figure 1:**
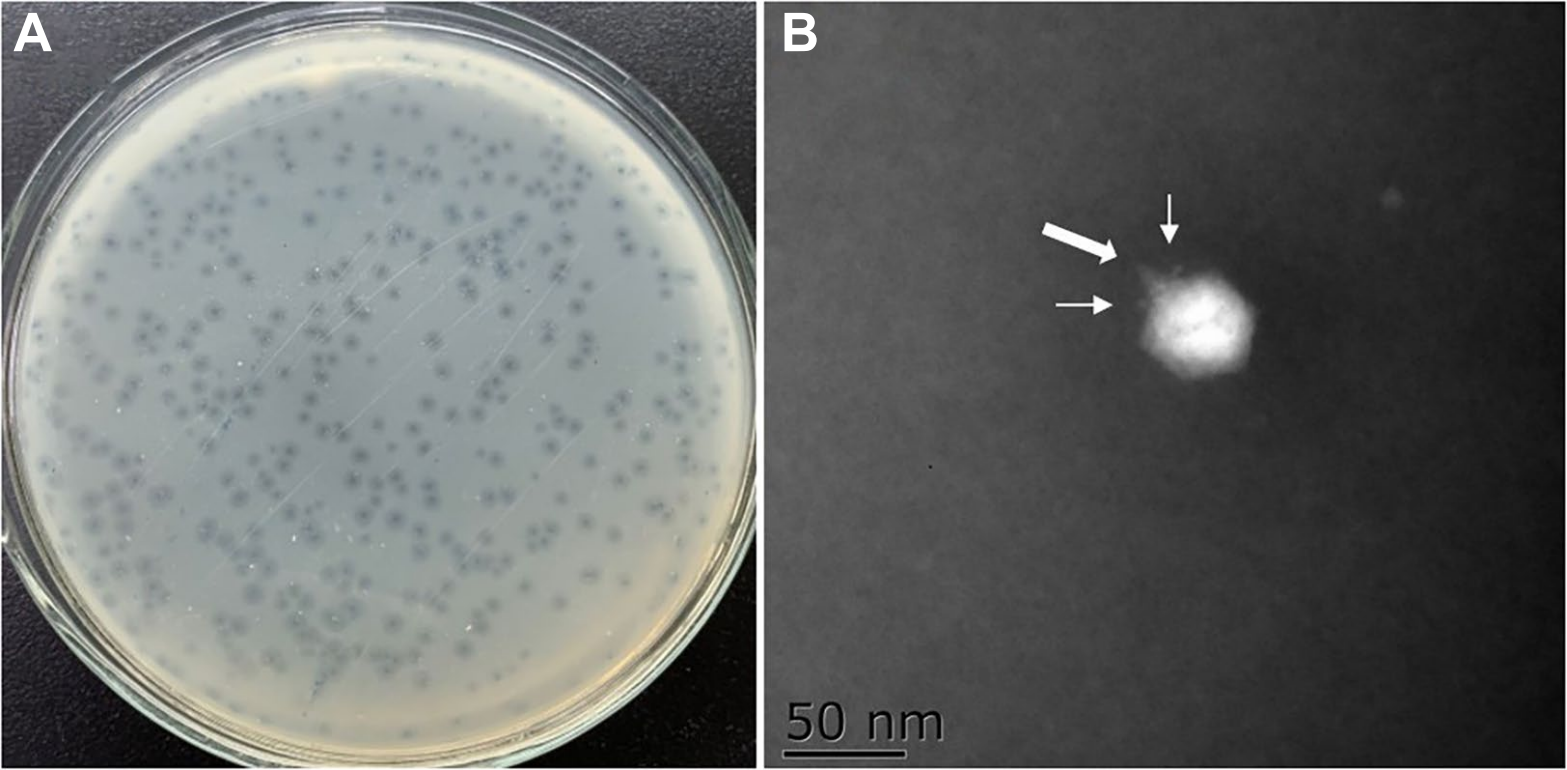
Morphology of the phage plaque and negative stained phage virions. **(A)** The plaques formed by phage OY1 on lawn of strain *Vibrio mimicus* CICC 21613. **(B)** Electron micrographs of phage OY1, the broad arrow indicate a tail and the narrow arrows indicate tail fibers.

Plaque-forming units (PFU) were observed in four of the six sewage samples collected from local aquatic product markets. A single isolate which produced clearest and largest plaque was picked and purified for each sample. The phage isolate OY1 showed the clearest and largest plaque, highest titer, absence of carrier state, and the broadest host range among the four isolates, and was thus been chosen for further characterization and evaluation of its potential as a biocontrol agent. It formed clear plaques of ~1.5 mm in diameter on the bacterial lawn of *V. mimicus* CICC 21613 with a turbid outer area (Figure 1A) and presented as an icosahedral particle of ~55 nm in diameter with a short tail of a ~8 nm and two tail fibers of ~2 nm under an electron microscope (Figure 1B). Morphological observation indicated that phage OY1 has a podovirus morphotype and belonged to the newly proposed family *Autographiviridae* based on the most recent taxonomy of this phage group (Turner et al., 2019), and also the high homology (>95%) of the whole genome of OY1 to other viruses classified in this family, i.e., vB_VpaP_KF1, vB_VpaP_KF2, and VP93 (see “Genome and Comparative Genomic Analysis” section).

### Biological Properties of Phage OY1

**Figure 2:**
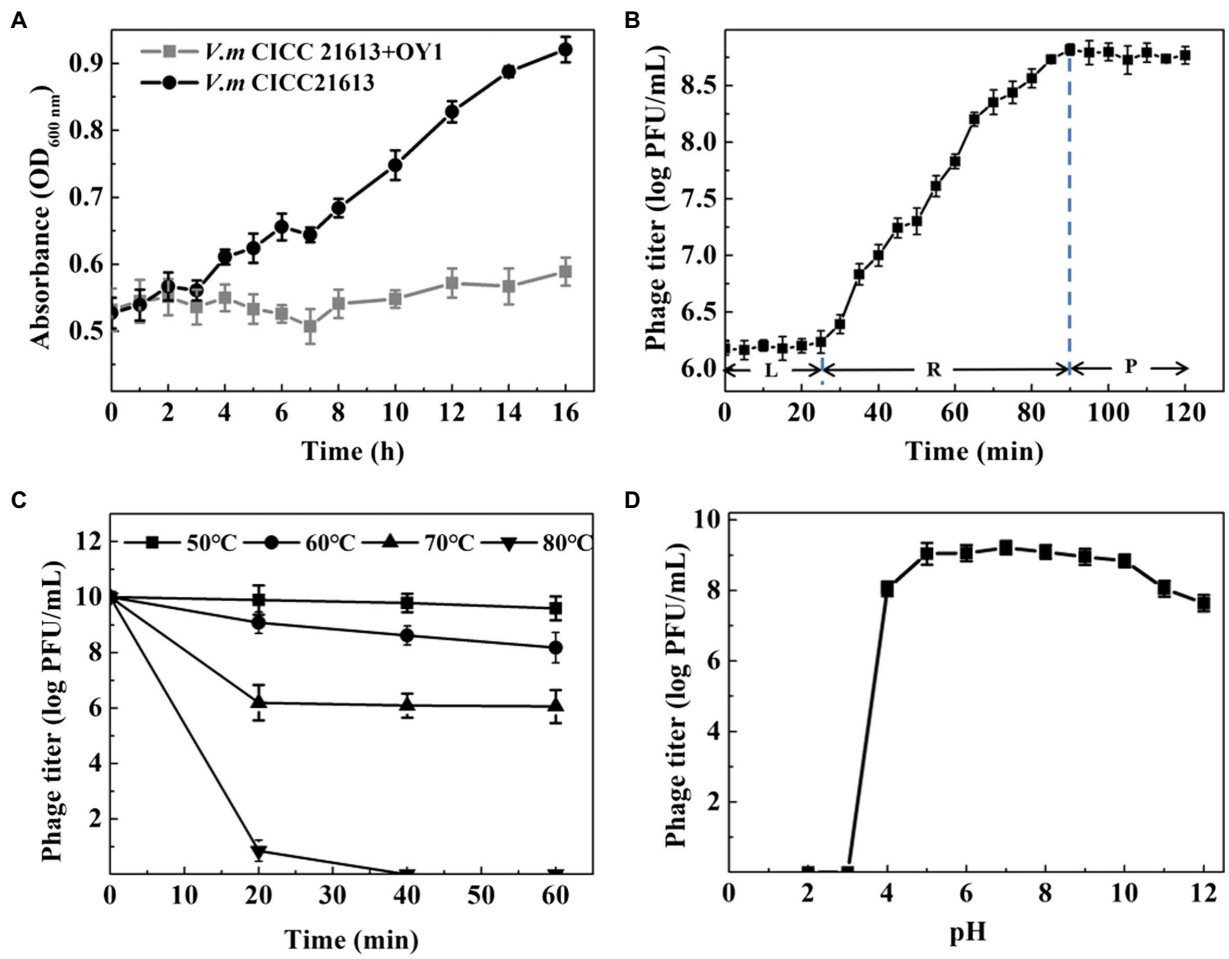
Biological properties of phage OY1. **(A)** Lytic activity of phage OY1 LB broth supplemented with 3% (w/v) NaCl. **(B)** One-step growth curve of phage OY1 in *V. mimicus* CICC 21613 at 37°C L, latent period; R, rise period; P, plateau period. Curves were determined in 3% LB broth at 37°C at multiplicity of infection (MOI) ratio of 0.01. **(C)** Temperature tolerance from 50°C to 80°C. **(D)** pH stability of phage OY1. Assays were performed in triplicate. Data are reported as the mean ± SD.

Bacterial lytic assay showed that OD_600 nm_ rose from 0.53 to 0.92 in a culture of *V. mimicus* CICC 21613 from 0 to 16 h post-inoculation, while retained the initial level when phage OY1 was inoculated into the culture (Figure 2A), indicating a high bacteriostatic efficiency of phage OY1 against *V. mimicus* CICC 21613 in LB culture.

One-step growth curve showed the latent period of the phage OY1 was about 25 min, followed by a 65 min rise period, and reached a plateau at about 90 min after being inoculated (Figure 2B). The final phage titer reached to ~10^8^ PFU/ml and the burst size was about 354 phage particles per cell.

Thermal resistance test showed that the titer of phage OY1 kept steady at 50°C for 1 h, while it decreased from ~10^10^ PFU/ml to ~10^9^ PFU/ml when kept at 60°C for 20 min and to ~10^8^ PFU/ml after 1 h. Phage titers decreased from ~10^10^ PFU/ml to ~10^6^ PFU/ml at 70°C when kept for 20 min or longer. Phages OY1 was almost completely inactivated after incubation at 80°C for 30 min (Figure 2C). The results of the study on pH stability showed that the phage OY1 was highly stable within the pH range of 5.0–10.0, while it was partially inactivated at pH values of 4.0–5.0 or 10.0–12.0, and almost completely inactivated at pH 3.0 and below (Figure 2D).

### Host Range of Phage OY1

Phage OY1 was capable of infecting 8 out of 11 *Vibrio* species and 24 out of 32 isolates covering over 75% of the tested strains (Table 1) including: 1 *V. aestuarianus* strain (VI13), 5 of 7 *V. alginolyticus* strains (VF221, VE13, VJ14, VG23, and VD31), 3 of 4 *V. azureus* strains (VJ31, VT44, and VK34), 1 of 2 *V. fluvialis* strains (CICC 21612), 1 of 2 *V. metschnikovii* strains (VN42), 2 of 2 *V. mimicus* strains (CICC 21613 and CICC 10474), 1 *V. natriegens* strain (VL22) and 10 *V. parahaemolyticus* strains (CICC 21617, Vp68, Vp76, Vp92, Vp101, Vp110, VJ34, VF13, VI52, and VD23) tested (EOP > 0). Two of seven *V. alginolyticus* strains (CICC 10484 and VH23), 1 of 4 *V. azureus* strains (VC32), the *V. cholera* strain CICC 23794, 1 of the 2 *V. fluvialis* strains (CGMCC 1.1609), the *V. harveyi* strain CGMCC 1.1609, 1 of the 2 *V. metschnikovii* strains (VM44), and the *V. owensii* strain VH51 showed resistance to the phage (Table 1).

### Genome and Comparative Genomic Analysis

**Figure 3:**
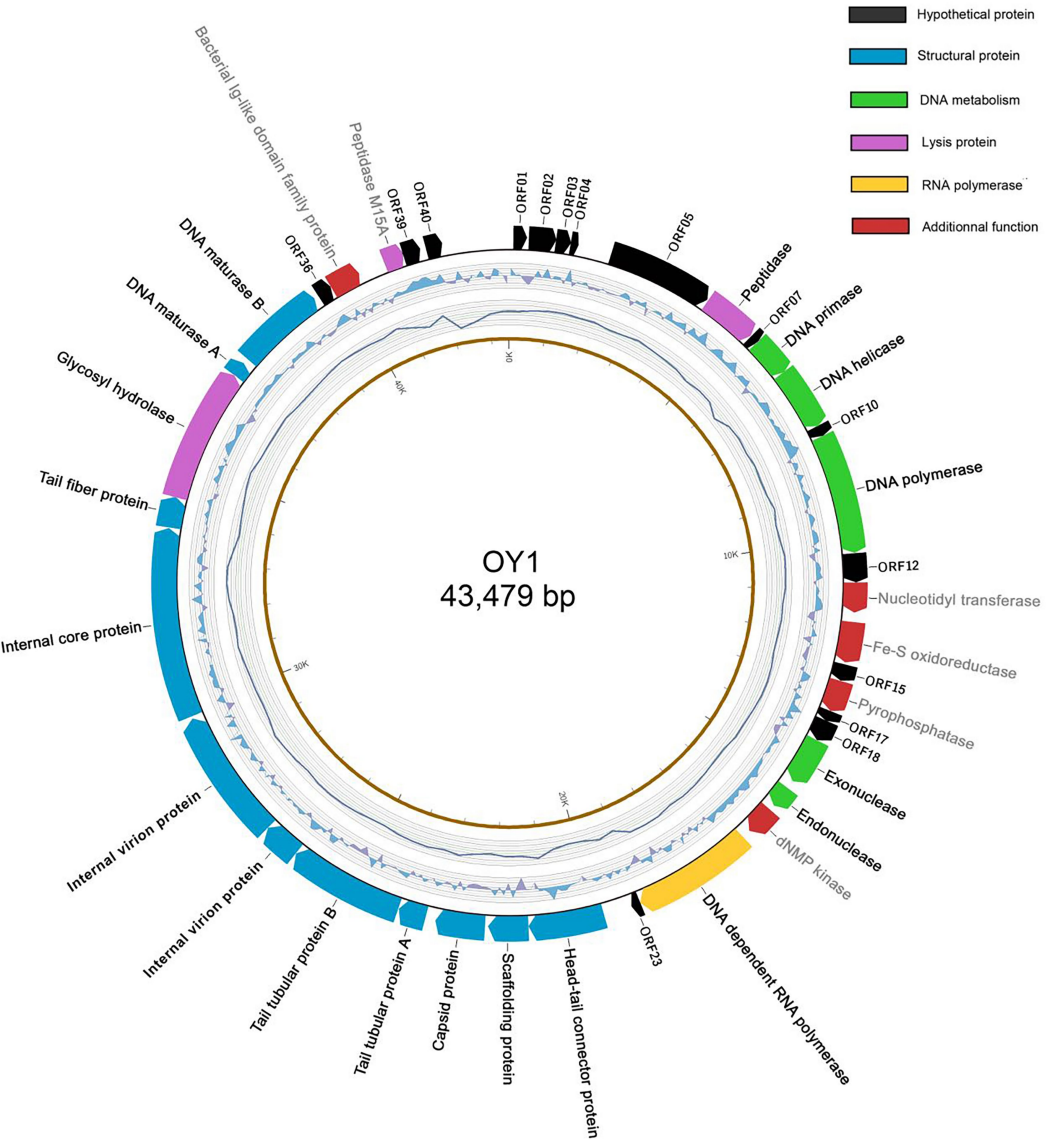
The genome structure of phage OY1. Arrows in clockwise indicate predicted protein-coding genes encoded on the Watson strand with gene names labeled on the external side (black: hypothetical protein; green: DNA metabolism related; yellow: RNA polymerase; blue: structural protein; purple: lysis protein; red: predicted proteins with additional functions). The second cycle (from outside) indicate GC skew (G − C/G + C in a 1-kb window and 0.1-kb incremental shift), value for the outer most line is 0.4 and −0.4 for the inner most. The third cycle indicates the sequencing coverage, the outer most line represents 9,500× and inner most represents 5,500×. The forth cycle is the physical map scaled in kbp.

The results revealed the phage OY1 genome consists of a circular double-stranded DNA of 43,479 bp with a GC content of 49.27% (Figure 3), encoding 40 ORFs with an average length of 966 bp, accounting for 88.84% of the total genome. Among all these ORFs, 17 (42.5%) were predicted by the protein–protein BLASTP against the five databases of NRDB, KEGG, COG, GO, and SWISS-PROT to include six structural proteins, nine proteins related to DNA manipulation and packaging, and two proteins related to cell lysis (Supplementary Table S1). In addition, eight proteins with known functions were predicted (with *E*-value <10^−5^) by a separate standard protein BLAST (BLASTP) in GenBank using the translated amino acid sequence as the query, including nucleotidyl transferase (ORF13), Fe-S oxidoreductase (ORF14), pyrophosphatase (ORF16), deoxynucleoside monophosphate kinase (dNMP kinase, ORF21), two internal virion proteins (ORF29 and ORF30), bacterial Ig-like domain family protein (ORF37), and peptidase M15A (ORF38). All the predicted protein-coding genes and ORFs were encoded on the same sense strand using ATG as the start codon (Figure 3; Supplementary Table S1, Supplementary material). However, the terminal codon usage was diverse, and TAA was found as the terminal codon in most cases (29 out of 40 ORFs). The tail tubular protein A, tail fiber protein, and five other hypothetical proteins used TAG as their terminal codon, while DNA maturase B and three other hypothetical proteins used TGA. No similar sequences encoding predicted toxins and virulence factors were found in the phage OY1 genome.

**Figure 4:**
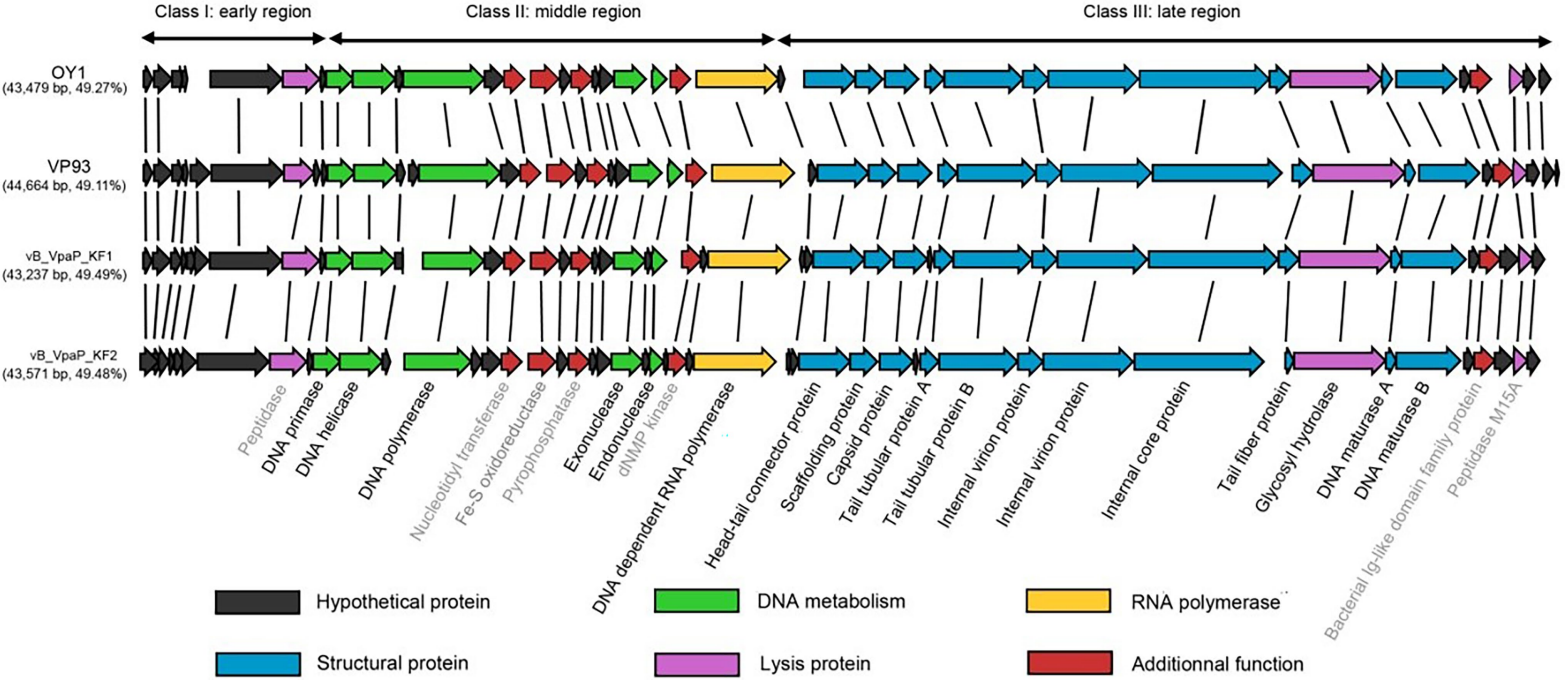
Comparison of the phage OY1 genome with three *Vibrio parahaemolyticus* phages, i.e., phages vB_VpaP_KF1 (accession no. MF754111), vB_VpaP_KF2 (accession no. MF754112) and VP93 (accession no. FJ896200). The predicted open reading frames (ORFs) are indicated by arrows. ORFs of unknown function are in black, predicted DNA metabolism ORFs in green, RNA polymerase in yellow, structural protein ORFs in blue, lysis protein ORFs in purple and ORFs with additional functions in red, respectively. Genes named in gray indicate the eight proteins that were predicted (with *E*-value <10^−5^) by a separate Standard Protein BLAST (blastp) in GenBank using translated amino acid sequence as the query.

The genome of phage OY1 can be divided into three genomic blocks (regions) which is in agreement with the typical characteristic of phiKMV-like viruses (Adriaenssens et al., 2011). The early region comprises a set of unknown genes (early genes) that are probably involved in host conversion and adaptation. Those early genes are reported to be transcribed immediately after infection, can protect the bacteriophage from the host defense systems, and able to adapt to the host (Roucourt and Lavigne, 2009). The middle region encompasses a gene cluster dealing with DNA metabolism and ends with the DNA-dependent RNA polymerase. The late region comprises genes coding for structural and lysis proteins, starting with a small gene (ORF23), followed by the genes coding for the head–tail connector protein (portal protein), scaffold protein, major capsid protein, and tubular tail proteins A and B, three internal virion proteins, tail fiber protein, hydrolase, the DNA maturase A and B, and peptidase (Figure 4).

A whole-genome blast was performed and phage OY1 showed a high homology (>95%) with genomes of three distinct species of the genus *Maculvirus*, i.e., *Vibrio virus KF1*, *Vibrio virus KF2* and *Vibrio virus VP93* (Bastías et al., 2010; Yu et al., 2018). Comparative genomic analysis showed that the protein-coding genes and their orders are highly conserved in *Maculvirus* phages (Figure 4).

### Inactivation of Sensitive Strains in FMEJ

**Figure 5:**
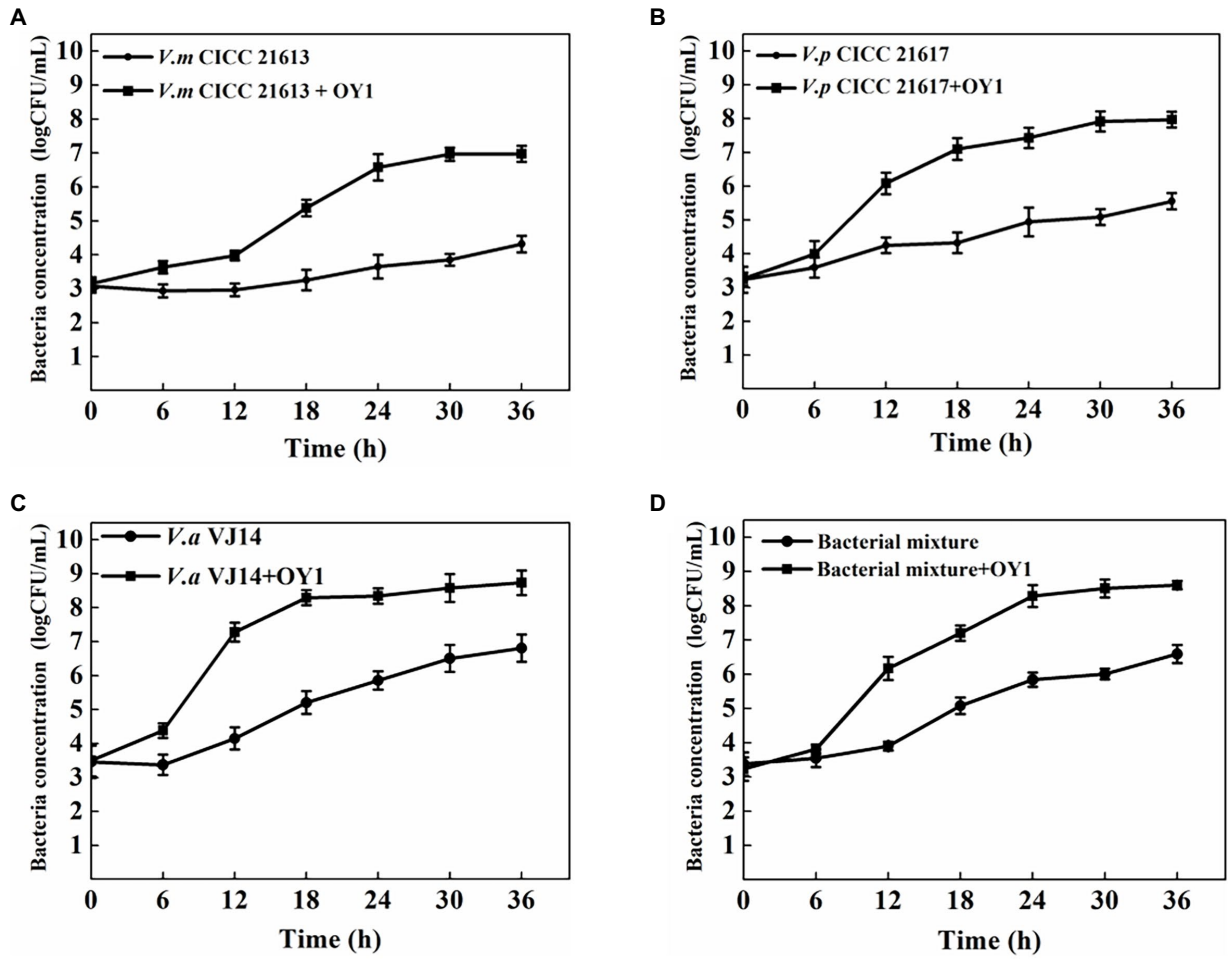
Inactivation of *V. mimicus* CICC 21613 **(A)**, *V. parahaemolyticus* CICC 21617 **(B)**, *V. alginolyticus* VJ14 **(C)**, and mixed bacteria **(D)** in FMEJ at 25°C. The test was performed in triplicates. Data are reported as the mean ± SD.

Considering that the phage OY1 was isolated from aquatic products and may probably be used as a food biocontrol agent, modeling using FMEJ as the substrate and single and mixed bacteria culture of *V. alginolyticus* VJ14, *V. parahaemolyticus* CICC 21617, and *V. mimicus* CICC 21613 as the target was performed to test the lytic activity of phage OY1 *in situ*. The bacterial concentration of *V. mimicus* CICC 21613 rose from 3.15 log CFU/ml to 6.97 log CFU/ml between 0 and 36 h incubation at 25°C. When infected with phage OY1 at 0 h, it rose slowly to 4.32 log CFU/ml after 36 h incubation, (Figure 5A). Similar inhibition was observed for *V. parahaemolyticus* CICC 21617, *V. alginolyticus* VJ14, and the mixed bacterial culture. The bacterial concentration reached 7.97 log CFU/ml, 8.73 log CFU/ml, and 8.60 log CFU/ml for *V. parahaemolyticus* CICC 21617, *V. alginolyticus* VJ14, and the mixed bacterial culture, respectively, after incubation at 25°C for 36 h. When phage OY1 was introduced, lower values of 5.55 log CFU/ml, 6.80 log CFU/ml, and 6.59 log CFU/ml were observed for *V. parahaemolyticus* CICC 21617 (Figure 5B), *V. alginolyticus* VJ14 (Figure 5C), and the mixed bacterial culture (Figure 5D), respectively, which was approximately two log below when compared to their cultures inoculated without phage OY1.

### Biofilm Prevention and Destruction

**Figure 6:**
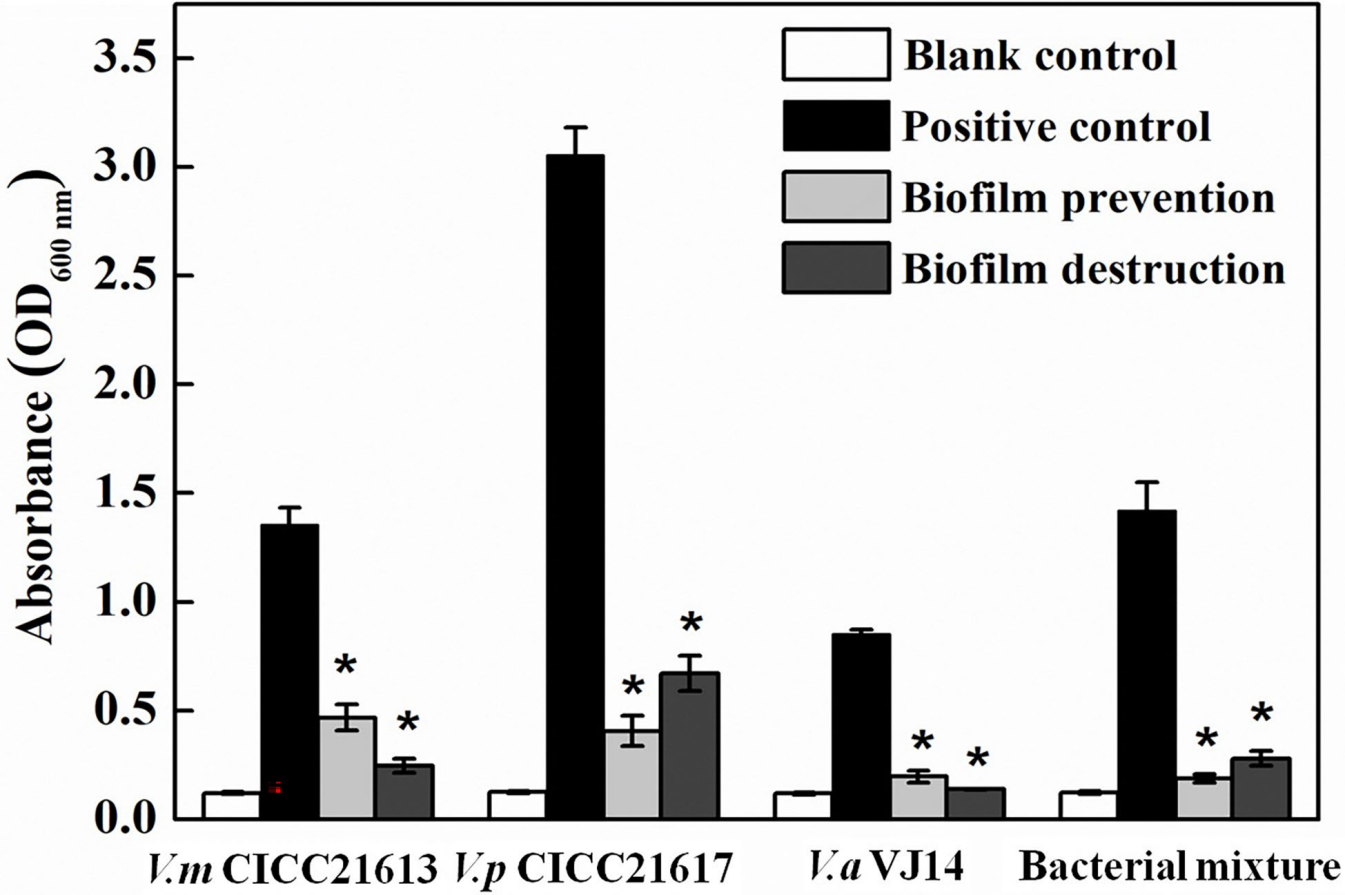
Biofilm prevention and destruction using phage OY1 against *V. mimicus* CICC 21613, *V. parahaemolyticus* CICC 21617, *V. alginolyticus* VJ14, and bacterial mixture. *indicates a significant difference (*p* < 0.01) compared with the blank control.

The microtiter plate test showed that the three *Vibrio* strains alone and their mixture were capable of forming biofilms in 3% LB. *Vibrio parahaemolyticus* CICC 21617 was found to produce more biofilms (OD_600 nm_ ~ 3.06) than the other two tested *Vibrio* isolates, i.e., *V. mimicus* CICC 21613 (OD_600 nm_ ~ 1.36), *V. alginolyticus* VJ14 (OD_600 nm_ ~ 0.85), and the mixed bacterial culture (OD_600 nm_ ~ 1.42; Figure 6). If co-cultured with phage OY1, biofilm formation was observed barely in the wells 24 h post-incubation at 37°C, for *V. alginolyticus* VJ14 (OD_600 nm_ ~ 0.20) and the mixed bacterial culture (OD_600 nm_ ~ 0.19). The other two strains, i.e., *V. mimicus* CICC 21613 and *V. parahaemolyticus* 21,617, formed filmy biofilms, wherein the absorbance of the culture at OD_600 nm_ was 0.41 and 0.47, respectively, comparing to 1.36 and 3.06 if cultures inoculated without phages.

To test the ability of the phage OY1 to destroy biofilms, the biofilms formed were treated with the phage suspension at 37°C for 4 h. Compared to the initial incubation which biofilms were formed, the absorbances of the final treated cultures at OD_600 nm_ was decreased from 1.36 to 0.25, 3.06 to 0.67, 0.85 to 0.14, and 1.42 to 0.28 for *V. mimicus* CICC 21613, *V. parahaemolyticus* CICC 21617, *V. alginolyticus* VJ14, and the mixed bacterial culture, respectively. The above observations indicate that phage OY1 could not only effectively disturbs the biofilms formed but also prevents biofilm formation.

## Discussion

Genomic and physical features of phage OY1 suggested a podovirus morphotype belonging to the newly proposed family *Autographiviridae* and the genus *Maculvirus* (Turner et al., 2019). Pairwise comparison of the complete genomic sequence of phage OY1 showed a high homology (>95%) with genomes of *V. parahaemolyticus* phages vB_VpaP_KF1, vB_VpaP_KF2, and VP93 (Bastías et al., 2010; Yu et al., 2018) which represented three distinct species of the genus, i.e., *Vibrio virus KF1*, *Vibrio virus KF2* and *Vibrio virus VP93*, respectively. Phage OY1 also had a similar genome architecture (gene content and synteny) as other *Autographiviridae* species, e.g., *Pantoea virus LIMElight*, *Pantoea virus LIMEzero*, *Pseudomonas virus LKA1*, and *Pseudomonas virus phiKMV* (Lavigne et al., 2003; Adriaenssens et al., 2011), however, the GC content of above species are significantly higher (54.0%–62.3%) than phage OY1 and the other *Maculvirus* phages (49.1%–49.5%, Figure 4).

The gene content and order are largely conserved among vB_VpaP_KF1, vB_VpaP_KF2, VP93, and OY1 (Figure 4). The similar genome architecture and the DNA similarity of these four *Vibrio* phages may indicate similar host specificity of species in this genus. *Vibrio virus KF1*, *Vibrio virus KF2*, *Vibrio virus OWB*, and *Vibrio virus VP93* were known to be isolated using *V. parahaemolyticus* as the host without exploring their host range (Bastías et al., 2010; Yu et al., 2018). Phage OY1 was isolated using the *V. mimicus* CICC 21613 as its host and showed lytic activity against all the 10 tested *V. parahaemolyticus* strains and 10 out of 22 tested other *Vibrio* strains, indicating a relative broad host range against *Vibrio* species. Host of phages in this group were possibly confined to *Vibrio* species. While in contrast, host range of species in other genera in the family *Autographiviridae* varied greatly. For example, phage LIMEzero was limited to the *Pantoea agglomerans* strain on which it was originally isolated (Adriaenssens et al., 2011), and phage LKA1 displayed an extremely narrow spectrum, lysing only two *Pseudomonas aeruginosa* strains among 74 tested (Ceyssens et al., 2006). However, the bacteriophage LIMElight and ɸKMV showed a relatively broader host range, the former infected two *Pantoea agglomerans* strains and showed an inhibitory effect on two *Pantoea stewartii* and two *Erwinia* isolates (Adriaenssens et al., 2011), and the latter showed high lytic activity in 10 out of 25 tested *Pseudomonas aeruginosa* strains (Lavigne et al., 2003).

Host range specificity of a bacteriophage is largely associated with its tail fiber proteins that allow the virus to recognize and bind to specific receptor sites on the surface of the host cell (Nguyen et al., 2001; Yoichi et al., 2005; Endersen et al., 2014). Scholl et al. (2001) reported an interesting case in which a bacteriophage encoded two different tail fiber proteins and could allow it to infect and replicate on two different strains. A single tail fiber protein was predicted (ORF32) and showed homology to several other *Vibrio* phages including *Vibrio virus KF1*, *Vibrio virus KF2*, *Vibrio virus OWB*, *Vibrio virus VP93* and et al., when blasted in GenBank (data not shown) indicating a possible host range of this group of bacteriophage which confines to *Vibrio* species. It would be interested to study the relationship between the sequence homology of tail fiber proteins and the host range variance using more phage isolates from different genera in this family.

Phage OY1 exhibited strong lytic activity against certain strains of four *Vibrio* species (Table 1), in which *V. parahaemolyticus* is a notable human pathogen, whereas the other three (*V. mimicus*, *V. alginolyticus*, and *V. metschnikovii*) are sporadically found in human infections (summarized in Thompson et al., 2004). Latent period and burst size are important factors to be considered in phages therapy; phages with high burst size and short latent period are usually more effective in lysing bacteria (Abedon and Culler, 2007; Abedon, 2021). Phage OY1 showed a higher burst size of about 316 phage particles per cell (Figure 2B), a strong lytic activity in addition to a wider host range compared to the other two *Vibrio* phages reported (Yin et al., 2019; Yang et al., 2020). Further, the phage OY1 was stable below 50°C (Figure 2C) and remained active in a wide pH range of 5.0–10.0 (Figure 2D). The wide thermal and pH tolerance indicate that the phage OY1 could be useful under different environmental conditions. Most importantly, no toxin gene were identified in the genome of phage OY1 (Supplementary Table S1). The above features brought the idea that it can be used as a candidate biocontrol agent (Hyman, 2019).

In addition, the ability of phage OY1 to prevent biofilm formation and to destroy biofilms was also tested, since *Vibrio* species form biofilms under different conditions and biofilm formation is part of the pathogenic lifestyles of the groups of bacteria tested in this study (Yildiz and Visick, 2009). Phage OY1 showed a strong ability to prevent biofilm formation and to destroy *Vibrio* biofilms (Figure 5). The above evidence indicates that the *Vibrio* bacteriophage OY1 could act as a potential biocontrol agent, either alone or as a component of a phage cocktail. Yin et al. (2019) recently isolated numbers of phages that specific to *V. parahaemolyticus* and tested the ability of three selected phages to prevent biofilm formation and to destroy established biofilms; it was found that the selected phages showed promise in preventing the development of biofilms while not effective in destroying established biofilms. Luo et al. (2016) isolated two phages which belonged to the family *Siphoviridae*; both phages were able to bring about two logs reduction in a *Vibrio harveyi* biofilm cell density after 24 h of phage treatment. However, biofilm prevention and reduction activities of the other four species in the genus *Maculvirus* against *Vibrio* species were not studied. Further experiments concerning the efficiency and safety of the phage OY1 alone, or a phage cocktail comprising OY1 under different field conditions are warranted, and studies on safety issues related to the use of the phage OY1 for aquaculture industry and seafood processing are needed.

It is interesting to find the hazy rings (haloes) surrounding the phage plaques of the phage OY1 over time (Figure 1A). Haloes were reported to be largely associated with depolymerases which are responsible for biofilm breakdown (Pires et al., 2016). In the genome of phage OY1, two peptidase (ORF06 and ORF38) and one glycosyl hydrolase (ORF33) were predicted, those three depolymerases were expected to be expressed during phage infection.

## Data Availability Statement

The datasets presented in this study can be found in online repositories. The names of the repository/repositories and accession number(s) can be found in the article/Supplementary Material.

## Author Contributions

LG and Z-QY conceived the study. MO executed the experiments and carried out the data analysis. LG, YL, and H-XL prepared the figures. YL, X-FZ, and S-QR analyzed the genome sequences. LG and YL wrote the draft of the manuscript. SG and YL revised the manuscript. HZ administrated the project. All authors contributed to the article and approved the submitted version.

## Funding

This study was supported by the National Natural Science Foundation of China (grant numbers 31371806 and 31901801), the Opening Fund of Jiangsu Key Laboratory for Food Quality and Safety-State Key Laboratory Cultivation Base (028074911709), China Yangzhou University Science and Technology Innovation Team (2016), and A Project Funded by the Priority Academic Program Development of Jiangsu Higher Education Institutions (PAPD).

## Conflict of Interest

The authors declare that the research was conducted in the absence of any commercial or financial relationships that could be construed as a potential conflict of interest.

## Publisher’s Note

All claims expressed in this article are solely those of the authors and do not necessarily represent those of their affiliated organizations, or those of the publisher, the editors and the reviewers. Any product that may be evaluated in this article, or claim that may be made by its manufacturer, is not guaranteed or endorsed by the publisher.

## Footnotes

^1^ http://www.repeatmasker.org/

^2^ http://tandem.bu.edu/trf/trf.html

^3^ http://blast.ncbi.nlm.nih.gov/
