# Supplementary material for: Isolation and Characterization of a Lytic Vibriophage OY1 and Its Biocontrol Effects Against Vibrio spp
Source: Front Microbiol. 2022 Apr 7;13:830692. doi: 10.3389/fmicb.2022.830692 (PMC9022663; doi:10.3389/fmicb.2022.830692)
Supplement: Supplementary file 1 [file Table_1.doc]

Table S1 Genome annotation of phage OY1.

| **Note** | **Database** | **Start codon** | **Start codon usage** | **End codon** | **Terminal codon usage** | **Strand** | **Length** | **Gene product** | **Function classification** | **Ontology** | **Blast hit with the highest max score (locus_tag, Query cover, E value, Ident )** |
| --- | --- | --- | --- | --- | --- | --- | --- | --- | --- | --- | --- |
| ORF01 | NR | 87 | ATG | 350 | TAA | + | 264 | Hypothetical protein | / | NA. | MF754112, *Vibrio parahaemolyticus* phage vB_VpaP_KF2 (KF2_010, 100%, 4e-119, 97%) |
| ORF02 | NR | 400 | ATG | 936 | TAA | + | 537 | Hypothetical protein | / | NA. | MF754112, *Vibrio parahaemolyticus* phage vB_VpaP_KF2 (KF2_011, 100%, 0.0, 96%) |
| ORF03 | NR | 939 | ATG | 1235 | TAG | + | 297 | Hypothetical protein | / | NA. | MF754112, *Vibrio parahaemolyticus* phage vB_VpaP_KF2 (KF2_012, 17%, 6e-07, 88%) |
| ORF04 | NR | 1244 | ATG | 1393 | TAA | + | 150 | Hypothetical protein | / | NA. | MF754111, *Vibrio parahaemolyticus* phage vB_VpaP_KF1 (KF1_027, 40% 1e-21 100%) |
| ORF05 | NR | 2085 | ATG | 4220 | TAA | + | 2136 | Hypothetical protein | / | NA. | MF754111, *Vibrio parahaemolyticus* phage vB_VpaP_KF1 (KF1_030, 99%, 0.0, 89%) |
| ORF06 | NR/GO | 4246 | ATG | 5349 | TAA | + | 1104 | Peptidase | Lysis | molecular function | MF754112, *Vibrio parahaemolyticu*s phage vB_VpaP_KF2 (KF2_017, 100%, 0.0, 94%) |
| ORF07 | NR | 5375 | ATG | 5539 | TGA | + | 165 | Hypothetical protein | / | NA. | MF754111, *Vibrio parahaemolyticus* phage vB_VpaP_KF1 (KF1_032, 100%, 1e-77, 99%) |
| ORF08 | NR | 5539 | ATG | 6351 | TAA | + | 813 | DNA primase | DNA manipulation | molecular function | MF754112, *Vibrio parahaemolyticus* phage vB_VpaP_KF2 (KF2_019, 100%, 0.0, 98%) |
| ORF09 | NR/GO | 6333 | ATG | 7613 | TAA | + | 1281 | DNA helicase | DNA manipulation | molecular function | MF754111, *Vibrio parahaemolyticus* phage vB_VpaP_KF1 (KF1_034, 100%, 0.0, 98%) |
| ORF10 | NR | 7613 | ATG | 7846 | TAG | + | 234 | Hypothetical protein | / | NA. | FJ896200, *Vibrio parahaemolyticu* phage VP93 (VPP93_gp12, 97%, 4e-113, 99%) |
| ORF11 | NR/GO | 7839 | ATG | 10265 | TAA | + | 2427 | DNA polymerase | DNA manipulation | molecular function | MF754111, *Vibrio parahaemolyticus* phage vB_VpaP_KF1 (KF1_036, 100%, 0.0, 95%) |
| ORF12 | NR | 10279 | ATG | 10857 | TAG | + | 579 | Hypothetical protein | / | NA. | FJ896200, *Vibrio parahaemolyticu* phage VP93 (VPP93_gp15, 100%, 1e-56, 70%) |
| ORF13 | NR | 10866 | ATG | 11462 | TAA | + | 597 | Nucleotidyl transferase* | Additional functions | molecular function | MF754112, *Vibrio parahaemolyticus* phage vB_VpaP_KF2 (KF2_025, 100%, 0.0, 95%) |
| ORF14 | NR | 11664 | ATG | 12479 | TAA | + | 816 | Fe-S oxidoreductase* | Additional functions | molecular function | MF754111, *Vibrio parahaemolyticus* phage vB_VpaP_KF1 (KF1_039, 100%, 0.0, 96%) |
| ORF15 | NR | 12525 | ATG | 12857 | TAA | + | 333 | Hypothetical protein | / | NA. | FJ896200, *Vibrio parahaemolyticu* phage VP93 (VPP93_gp18, 100%, 5e-159, 98%) |
| ORF16 | NR/  Swiss-Prot | 12869 | ATG | 13498 | TAA | + | 630 | Pyrophosphatase* | Additional functions | molecular function | MF754111, *Vibrio parahaemolyticus* phage vB_VpaP_KF1 (KF1_041, 100%, 0.0, 97%) |
| ORF17 | NR | 13498 | ATG | 13707 | TAA | + | 210 | Hypothetical protein | / | NA. | MF754112, *Vibrio parahaemolyticus* phage vB_VpaP_KF2 (KF2_029, 100%, 1e-102, 99%) |
| ORF18 | NR | 13717 | ATG | 14142 | TAA | + | 426 | Hypothetical protein | / | NA. | MF754112, *Vibrio parahaemolyticus* phage vB_VpaP_KF2 (KF2_030, 100%, 0.0, 97%) |
| ORF19 | NR/GO | 14145 | ATG | 15095 | TAA | + | 951 | Exonuclease | DNA manipulation | molecular function | MF754111, *Vibrio parahaemolyticus* phage vB_VpaP_KF1 (KF1_044, 99%, 0.0, 98%) |
| ORF20 | NR/GO | 15275 | ATG | 15715 | TAA | + | 441 | Endonuclease | DNA manipulation | molecular function | MF754112, *Vibrio parahaemolyticus* phage vB_VpaP_KF2 (KF2_033, 100%, 0.0, 98%) |
| ORF21 | NR | 15831 | ATG | 16415 | TAA | + | 585 | dNMP kinase* | Additional functions | molecular function | FJ896200, *Vibrio parahaemolyticu* phage VP93 (VPP93_gp24, 99%, 0.0, 96%) |
| ORF22 | NR/  Swiss-Prot/  GO | 16604 | ATG | 19054 | TAA | + | 2451 | DNA dependent RNA polymerase | DNA manipulation | molecular function | FJ896200, *Vibrio parahaemolyticu* phage VP93 (VPP93_gp25, 100%, 0.0, 97%) |
| ORF23 | NR | 19054 | ATG | 19239 | TAG | + | 186 | Hypothetical protein | / | NA. | MF754111, *Vibrio parahaemolyticus* phage vB_VpaP_KF1 (KF1_004, 100%, 1e-84, 98%) |
| ORF24 | NR/  Swiss-Prot | 19823 | ATG | 21355 | TAA | + | 1533 | Head-tail connector protein | DNA packaging | cellular component | MF754111, *Vibrio parahaemolyticus* phage vB_VpaP_KF1 (KF1_006, 100%, 0.0, 98%) |
| ORF25 | NR | 21355 | ATG | 22170 | TAA | + | 816 | Scaffolding protein | Structural protein | cellular component | MF754111, *Vibrio parahaemolyticus* phage vB_VpaP_KF1 (KF1_007, 100%, 0.0, 98%) |
| ORF26 | NR/  Swiss-Prot | 22235 | ATG | 23233 | TAA | + | 999 | Capsid protein | Structural protein | cellular component | FJ896200, *Vibrio parahaemolyticu* phage VP93 (VPP93_gp29, 100%, 0.0, 98%) |
| ORF27 | NR | 23439 | ATG | 23999 | TAG | + | 561 | Tail tubular protein A | Structural protein | cellular component | FJ896200, *Vibrio parahaemolyticu* phage VP93 (VPP93_gp30, 100%, 0.0, 99%) |
| ORF28 | NR/  Swiss-Prot | 24009 | ATG | 26354 | TAA | + | 2346 | Tail tubular protein B | Structural protein | cellular component | FJ896200, *Vibrio parahaemolyticu* phage VP93 (VPP93_gp31, 100%, 0.0, 92%) |
| ORF29 | NR | 26364 | ATG | 27113 | TAA | + | 750 | Internal virion protein* | Additional functions | cellular component | MF754111, *Vibrio parahaemolyticus* phage vB_VpaP_KF1 (KF1_012, 100%, 0.0, 98%) |
| ORF30 | NR | 27123 | ATG | 29801 | TAA | + | 2679 | Internal virion protein* | Additional functions | cellular component | FJ896200, *Vibrio parahaemolyticu* phage VP93 (VPP93_gp33, 100%, 0.0, 96%) |
| ORF31 | NR | 29852 | ATG | 33706 | TAA | + | 3855 | Internal core protein | Structural protein | cellular component | MF754111, *Vibrio parahaemolyticus* phage vB_VpaP_KF1 (KF1_014, 100%, 0.0, 98%) |
| ORF32 | NR | 33727 | ATG | 34338 | TAG | + | 612 | Tail fiber protein | Structural protein | cellular component | MF754111, *Vibrio parahaemolyticus* phage vB_VpaP_KF1 (KF1_015, 100%, 0.0, 98%) |
| ORF33 | NR/GO | 34347 | ATG | 37079 | TAA | + | 2733 | Glycosyl hydrolase | Lysis | molecular function | MF754111, *Vibrio parahaemolyticus* phage vB_VpaP_KF1 (KF1_016, 100%, 0.0, 97%) |
| ORF34 | NR | 37089 | ATG | 37388 | TAA | + | 300 | DNA maturase A | DNA manipulation | cellular component | MF754112, *Vibrio parahaemolyticus* phage vB_VpaP_KF2 (KF2_003, 100%, 2e-142, 98%) |
| ORF35 | NR/  Swiss-Prot | 37513 | ATG | 39318 | TGA | + | 1806 | DNA maturase B | DNA manipulation | cellular component | FJ896200, *Vibrio parahaemolyticu* phage VP93 (VPP93_gp38, 100%, 0.0, 99%) |
| ORF36 | NR | 39423 | ATG | 39722 | TAG | + | 300 | Hypothetical protein | / | NA. | MF754112, *Vibrio parahaemolyticus* phage vB_VpaP_KF2 (KF2_005, 100% 9e-146 98%) |
| ORF37 | NR | 39731 | ATG | 40345 | TAA | + | 615 | Bacterial Ig-like domain family protein* | Additional functions | biological process | MF754111, *Vibrio parahaemolyticus* phage vB_VpaP_KF1 (KF1_020, 100%, 0.0, 98%) |
| ORF38 | NR | 40908 | ATG | 41321 | TGA | + | 414 | Peptidase M15A* | Additional functions | molecular function | MF754111, *Vibrio parahaemolyticus* phage vB_VpaP_KF1 (KF1_022, 100%, 0.0, 97%) |
| ORF39 | NR | 41314 | ATG | 41670 | TAA | + | 357 | Hypothetical protein | / | NA. | MF754111, *Vibrio parahaemolyticus* phage vB_VpaP_KF1 (KF1_023, 100%, 2e-177, 99%) |
| ORF40 | NR | 41789 | ATG | 42124 | TGA | + | 336 | Hypothetical protein | / | NA. | FJ896200, *Vibrio parahaemolyticu* phage VP93 (VPP93_gp43, 58%, 2e-62, 91%) |
